# The diagnostic and prognostic value of APRI and de Ritis ratio in intrahepatic cholestasis of pregnancy

**DOI:** 10.1186/s12884-025-08056-3

**Published:** 2025-08-28

**Authors:** Ebu Bekir Sıddık Yılmaz, Erkan Sağlam, Serenat Yalcin, Mustafa Raşit Özler

**Affiliations:** Department of Obstetrics and Gynecology, Department of Perinatology, Bursa City Hospital, Bursa, Turkey

**Keywords:** APRI, De ritis ratio, Intrahepatic cholestasis of pregnancy, Diagnosis, Severity, Non-invasive marker

## Abstract

**Objective:**

Intrahepatic cholestasis of pregnancy (ICP) is associated with adverse perinatal outcomes. Accurate and timely diagnosis is essential to minimize maternal and fetal risks. The standard diagnostic method—measurement of fasting serum bile acids (BA)—poses challenges due to limited availability, high cost, and delayed results. This study aimed to evaluate the diagnostic and prognostic utility of the aspartate aminotransferase to platelet ratio index (APRI) and the De Ritis ratio in ICP.

**Methods:**

This retrospective case-control study included 238 pregnant women, categorized into three groups: mild ICP (*n* = 62), severe ICP (*n* = 57), and healthy controls (*n* = 119). Demographic, biochemical, and perinatal outcomes were compared among the groups. APRI and De Ritis ratios were calculated and analyzed using receiver operating characteristic (ROC) curves and correlation analyses.

**Results:**

APRI levels were significantly elevated in ICP patients and demonstrated strong diagnostic performance for identifying ICP (cut-off: 0.295; AUC = 0.842). The De Ritis ratio was significantly lower in both ICP groups compared with controls (cut-off: 1.097; AUC = 0.770). APRI also correlated with disease severity (cut-off: 0.615 for severe ICP; AUC = 0.868). Higher APRI values were associated with increased neonatal intensive care unit (NICU) admissions and lower gestational age and birth weight.

**Conclusions:**

APRI and De Ritis ratios are simple, inexpensive, and non-invasive markers that may aid in the diagnosis and severity assessment of ICP. They could serve as useful alternatives in clinical settings where BA testing is limited or unavailable.

## Introduction

Intrahepatic cholestasis of pregnancy (ICP), the most common liver disorder during pregnancy, is characterized by elevated liver function tests—such as aspartate aminotransferase (AST), alanine aminotransferase (ALT), and/or serum bile acids (BA)—accompanied by pruritus that cannot be attributed to other causes. It typically occurs in the second and third trimesters of pregnancy, with an incidence of 0.5–1.0% [1–6].

ICP has been associated with adverse perinatal outcomes, including preterm birth, neonatal respiratory distress, meconium-stained amniotic fluid, and even stillbirth. In some cases, cholestasis may persist postpartum, potentially progressing to liver fibrosis or cirrhosis. Therefore, timely and accurate diagnosis of ICP, followed by early treatment initiation, is essential to protect both maternal and fetal health [6].

Furthermore, recent evidence suggests that certain pregnancy-related disorders, such as preeclampsia, may have lingering effects on maternal organ systems—particularly renal and cardiovascular—even in the early postpartum period. Ayguler and Anik Ilhan [7] observed that hypertension and proteinuria persisted in a substantial proportion of preeclamptic women at six weeks postpartum, underlining the systemic and ongoing nature of such conditions beyond delivery. This highlights the importance of identifying reliable and accessible biomarkers—like APRI and the De Ritis ratio—not only for timely diagnosis but also for the longitudinal monitoring of hepatic involvement in pregnancy-related pathologies.

Measurement of elevated serum BA—the gold standard for ICP diagnosis—presents several limitations. It requires specialized laboratory methods, is time-consuming, not universally available, and often results in reporting delays. Consequently, recent studies have explored the utility of blood-based markers for predicting ICP [5].

One such marker is the AST to Platelet Ratio Index (APRI), a noninvasive formula calculated as (AST/upper normal limit) ÷ platelet count × 100. APRI was originally developed to assess liver fibrosis and cirrhosis in patients with chronic hepatitis C [8]. Another relevant marker is the De Ritis ratio, defined as the AST-to-ALT ratio, which has traditionally been used as a biochemical indicator of liver injury and to differentiate among hepatic disorders [9].

In recent years, an increasing number of studies have investigated the utility of APRI as a noninvasive marker in pregnancy-related conditions, including ICP. Several authors have reported significantly elevated APRI levels in pregnant women with ICP, particularly during the third trimester. These findings suggest that APRI may function as an early indicator of ICP and may be associated with disease severity and adverse perinatal outcomes [4, 10–12].

In the present study, we aimed to assess the predictive value of APRI for the diagnosis and severity of ICP, its correlation with perinatal outcomes, and to determine whether APRI and the De Ritis ratio could serve as practical and accessible diagnostic tools in routine obstetric care.

## Materials and methods

This retrospective case-control study analyzed hospital records of pregnant women diagnosed with ICP between January 1, 2022, and April 1, 2025. The study population was categorized into three groups: mild ICP (BA 10–39 µmol/L, *n* = 62), severe ICP (BA ≥ 40 µmol/L, *n* = 57), and healthy pregnant women (BA < 10 µmol/L, *n* = 119). Disease severity was classified according to fasting serum bile acid concentrations (mild: 10–39 µmol/L; severe: ≥40 µmol/L).

Pregnant women diagnosed with ICP—defined as having a total BA level ≥ 10 µmol/L during pregnancy—with available third-trimester complete blood count, AST, ALT, platelet count (PLT), and neonatal outcome data were included. Healthy pregnant women without any history or signs of hepatobiliary disease or liver dysfunction were selected as controls.

Pruritus symptoms were evaluated based on medical records and clinical notes. The onset time and distribution of pruritus were reviewed whenever documented. No evidence of pruritus was found in the control group. In several ICP cases, medical records indicated that pruritus had preceded the elevation of serum bile acids or liver enzymes.

Gestational diabetes mellitus (GDM) status was determined retrospectively based on medical records. Diagnosis in the clinical setting had been established according to IADPSG criteria using a 75 g oral glucose tolerance test (OGTT) performed at 24–28 weeks of gestation. Women with GDM were excluded from the control group.

Exclusion criteria were as follows: diagnosis of preeclampsia (PE), HELLP syndrome (Hemolysis, Elevated Liver Enzymes, and Low Platelet Count), other hepatobiliary disorders, pre-existing liver disease (e.g., viral or autoimmune hepatitis), history of biliary tract disease or gallstones, use of hepatotoxic medications, smoking, alcohol or substance abuse, and incomplete laboratory records. Multiple pregnancies were also excluded to avoid confounding effects on maternal liver function and neonatal outcomes.

Hepatobiliary ultrasonography was performed only when clinically indicated (e.g., elevated liver enzymes or pruritus) to rule out other hepatic conditions such as gallstones or liver masses. Healthy controls were selected from low-risk pregnancies with normal liver function and no clinical suspicion of liver or biliary tract disease. All participants were from the same geographic and ethnic background, thereby minimizing population-based variability.

Demographic and clinical data including maternal age, gestational age, body mass index (BMI, kg/m²)—which was included as a baseline variable to assess its potential confounding effect on liver function parameters—obstetric history, complete blood count (hemogram), AST, ALT, platelet count (PLT), fasting bile acid levels, delivery week, and neonatal outcomes (including birth weight, 1st- and 5th-minute Apgar scores, and neonatal intensive care unit [NICU] admission) were retrieved from hospital records.

Serum total bile acids were assessed from the laboratory records. All measurements had been performed using an enzymatic colorimetric assay (Diazyme Laboratories, Poway, CA, USA). All samples were analyzed in the same central laboratory using the same method.

APRI was calculated using the following formula: (AST/upper normal limit) ÷ PLT count (10⁹/L) × 100. The upper reference limit for AST was defined as 32 U/L, based on the standard cut-off value used by our hospital’s central laboratory. This value reflects a locally validated threshold established according to national guidelines and internal quality control protocols.

Data analysis was conducted using IBM SPSS Statistics for Windows, version 26.0 (IBM Corp., Armonk, NY, USA).

Patients diagnosed with ICP had been treated with ursodeoxycholic acid (UDCA), and the timing of delivery had been individualized based on disease severity. Information on close fetal monitoring was obtained from medical records. According to these records, most patients underwent weekly nonstress tests (NST) and biweekly biophysical profile (BPP) assessments, while umbilical artery Doppler was performed only in cases with additional maternal or fetal risk factors. For patients with gestational diabetes mellitus (GDM), dietary modifications and medical nutrition therapy had been prescribed, and insulin therapy was initiated when glycemic targets were not achieved, according to standard clinical protocols.

Descriptive statistics were presented as mean ± standard deviation for normally distributed continuous variables, and as median (minimum–maximum) for variables not normally distributed. Categorical variables were expressed as numbers (n) and percentages (%).

To assess the distribution of variables, coefficient of variation, histograms, skewness, and kurtosis values were examined. The Kolmogorov–Smirnov test was used to statistically assess normality.

For comparisons among the three groups, the Kruskal–Wallis test was used for continuous variables that did not follow a normal distribution. Post-hoc pairwise comparisons were conducted using Tamhane’s T2 test. Categorical variables were compared using the Chi-square test or Fisher’s exact test, as appropriate.

The diagnostic performance of APRI was assessed using receiver operating characteristic (ROC) curve analysis. The correlation between APRI and other variables was evaluated using Spearman correlation analysis. A p-value of < 0.05 was considered statistically significant in all analyses.

## Results

### Demographic characteristic

A total of 238 pregnant women were included in the study: 62 with mild ICP, 57 with severe ICP, and 119 healthy controls. There were no statistically significant differences among the groups in terms of maternal age, BMI, gravida, or gestational age at the time of ICP diagnosis. The prevalence of gestational diabetes mellitus (GDM) was also comparable across the groups (*p* > 0.05) (Table 1).

**Table 1:** Comparison of demographic characteristics and birth outcomes among pregnant women with mild and severe intrahepatic cholestasis of pregnancy (ICP) and healthy pregnant women

Variable	Mild ICP (*n* = 62)	Severe ICP (*n* = 57)	Healthy (*n* = 119)	*p* value	Post-hoc Difference
Age (years)	28 (19–42)	30 (21–42)	29 (18–44)	0.357	–
BMI (kg/m²)	29 (18–50)	28 (18–40)	27 (18–46)	0.134	–
Gravida	2 (1–7)	2 (1–8)	2 (1–5)	0.652	–
Diagnosis week	33 (24–38)	33 (17–40)	34 (26–40)	0.159	–
Week of birth	37 (28–40)	36 (32–40)	39 (27–41)	**< 0.001**	3 > 1, 3 > 2
Birth weight (g)	2860 (955–4540)	2895 (2140–3640)	3340 (1020–5150)	**< 0.001**	3 > 1, 3 > 2
Apgar 1 st min.	9 (0–9)	7 (4–9)	9 (4–9)	**< 0.001**	1 > 2, 3 > 1, 3 > 2
Apgar 5th min.	10 (0–10)	9 (6–10)	10 (7–10)	**< 0.001**	3 > 1, 3 > 2
Type of delivery (NVD/C/S)	25 (40.3%)/37 (59.7%)	23 (40.4%)/34 (59.6%)	54 (45.4%)/65 (54.6%)	0.734	–
NICU admission (Yes/No)	14 (22.6%)/47 (75.8%)/1 (1.6%)	25 (43.9%)/32 (56.1%)	2 (1.7%)/117 (98.3%)	< 0.001	–
**GDM**,** n (%)**	6 (9.7%)	5 (8.8%)	10 (8.4%)	0.937	–

Data are presented as median (min–max) or number (percentage), as appropriate. Post-hoc comparisons were conducted using Tamhane’s T2 test

*BMI* Body mass index, *kg/m*^*2*^ kilograms per square meter, *NVD* Normal vaginal delivery, *C/S* Caesarean section, *NICU* Neonatal intensive care unit

*p* values obtained by Kruskal–Wallis test for continuous variables and Pearson’s Chi-square or Fisher’s exact test for categorical variables

### Perinatal outcomes

Gestational age at delivery and birth weight were significantly lower in both the mild and severe ICP groups compared with the healthy pregnancy group (*p* < 0.05). First- and fifth-minute Apgar scores were also reduced in the ICP groups, with the lowest values observed in the severe ICP group (*p* < 0.05). Although some very low Apgar scores (e.g., 0 or 4) were noted, these were infrequent and did not reflect a consistent trend. There was no statistically significant difference in the mode of delivery among the groups. However, NICU admission was more frequent in the severe ICP group (*p* < 0.05). The interval between diagnosis and delivery ranged from 1 to 7 weeks, depending on disease severity and clinical management. No significant differences were observed in indications for cesarean section or in hepatobiliary ultrasonographic findings (*p* > 0.05) (Table 1).

### Laboratory parameters

White blood cell (WBC) count was significantly lower in the severe ICP group compared with the healthy controls (*p* < 0.05). ALT and AST levels were elevated in both ICP groups compared with the healthy group and were significantly higher in the severe ICP group than in the mild group (*p* < 0.05). Platelet counts were lower in the severe ICP group compared with the mild group (*p* < 0.05). Total bilirubin was elevated in the severe ICP group, and direct bilirubin was increased in both ICP groups relative to the control group (*p* < 0.05) (Table 2).

**Table 2:** Comparison of laboratory parameters, APRI index, and de Ritis ratio among pregnant women with mild and severe intrahepatic cholestasis of pregnancy (ICP) and healthy pregnant women

Parameter	Mild ICP (*n* = 62)	Severe ICP (*n* = 57)	Healthy (*n* = 119)	*p* value	Post-hoc Difference
WBC (×10^3^/µL)	9.7 (0.01–20.1)	9.2 (6.2–16.0)	10.1 (0.01–23.0)	**0.038**	**3 > 2**
PLT (×10^3^/µL)	245 (141–462)	226 (63–502)	237 (29.2–450)	**0.041**	**1 > 2**
ALT (U/L)	28.5 (5–487)	105 (14–2044)	12 (5–139)	**< 0.001**	**1 > 3**,** 2 > 3**,** 2 > 1**
AST (U/L)	29.5 (9–259)	74 (20–926)	16 (9–89)	**< 0.001**	**1 > 3**,** 2 > 3**,** 2 > 1**
Total Bilirubin (mg/dL)	0.4 (0.1–55.0)	0.5 (0.1–4.3)	0.2 (0.1–1.0)	**< 0.001**	**2 > 3**
Direct Bilirubin (mg/dL)	0.1 (0.07–1.5)	0.2 (0.07–3.3)	0.1 (0.04–0.6)	**< 0.001**	**1 > 3**,** 2 > 3**
Fasting Bile Acids (µmol/L)	17.5 (10–38)	71.4 (40.4–181.0)	2.9 (0.3–9.9)	**< 0.001**	**1 > 3**,** 2 > 3**,** 2 > 1**
APRI	0.3 (0.08–4.6)	1.1 (0.2–12.0)	0.2 (0.08–0.80)	**< 0.001**	**1 > 3**,** 2 > 3**,** 2 > 1**
De Ritis Ratio	0.9 (0.1–3.0)	0.7 (0.3–3.0)	1.3 (0.4–3.1)	**< 0.001**	**3 > 1**,** 3 > 2**,** 1 > 2**

Data are presented as median (min–max)

*p* values were calculated using the Kruskal–Wallis test for group comparisons; post-hoc comparisons were conducted using Tamhane’s T2 test

*WBC* White blood cell count, *PLT* Platelet count, *ALT* Alanine aminotransferase, *AST* Aspartate aminotransferase, *APRI* Aspartate aminotransferase-to-platelet ratio index

### Correlation analyses

**Fig. 1:**
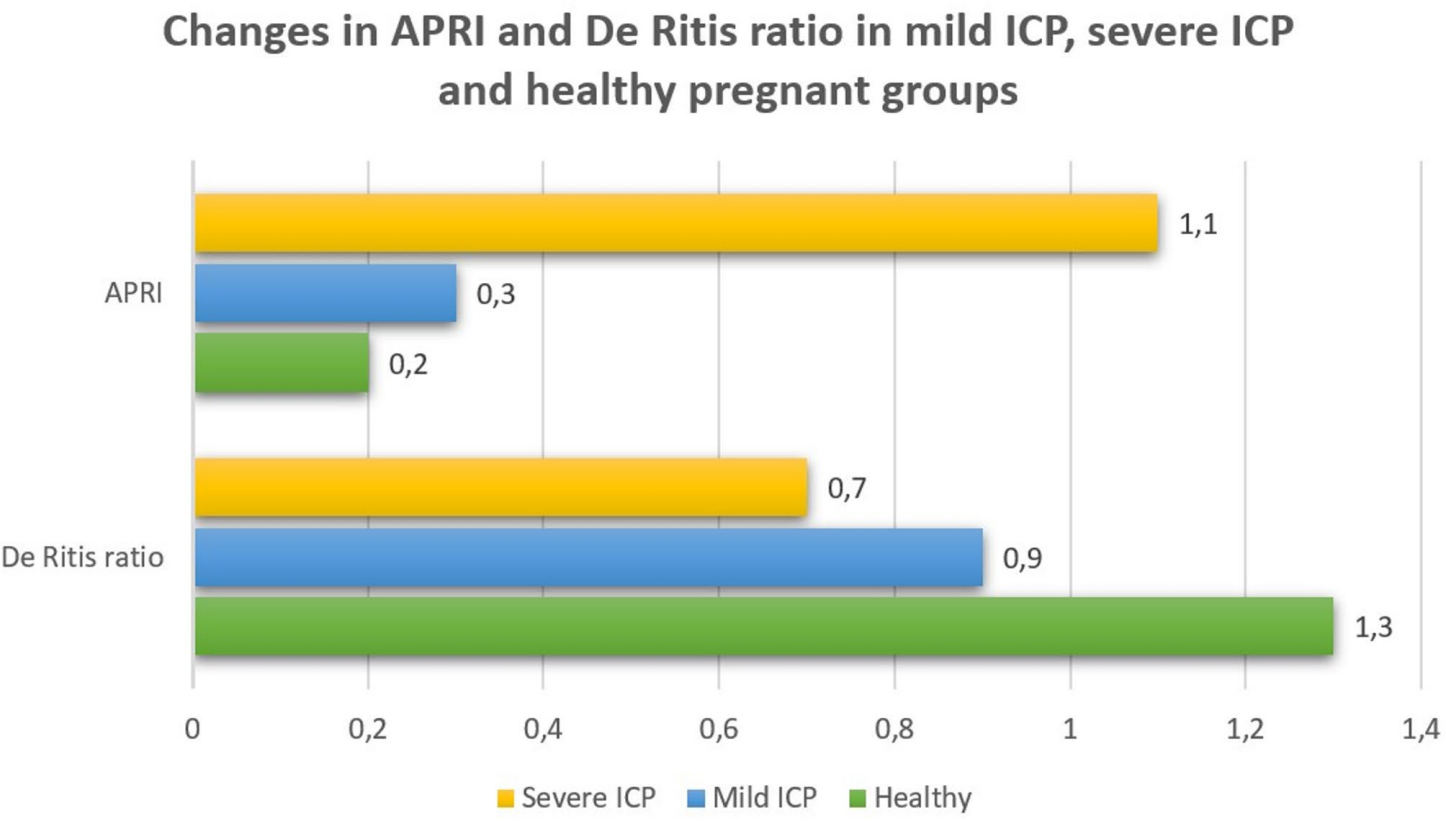
Changes in APRI and De Ritis ratio in mild ICP, severe ICP and healthy pregnant groups. This bar chart illustrates the distribution of Aspartate Aminotransferase Platelet Ratio Index (APRI) and De Ritis ratio (AST/ALT) among three groups: healthy pregnant women, patients with mild intrahepatic cholestasis of pregnancy (ICP), and those with severe ICP. The x-axis represents the index values, while the y-axis indicates the parameter (APRI or De Ritis ratio). Mean values are labeled directly on each bar for clarity. The graph demonstrates elevated APRI levels in severe ICP compared with mild ICP and healthy groups, whereas the De Ritis ratio is lower in severe ICP and highest among healthy pregnant women

Fasting BA and APRI values were significantly higher in both mild and severe ICP groups compared with the healthy group and were also higher in the severe group than in the mild group (*p* < 0.05). In contrast, the De Ritis ratio was significantly higher in the healthy group compared with both ICP groups, and higher in the mild group than in the severe group (*p* < 0.05) (Fig. 1; Table 2).

In the mild ICP group, APRI showed a positive correlation with NICU admission and a negative correlation with the De Ritis ratio and birth weight. In the severe ICP group, APRI was positively correlated with fasting BA and NICU admission, and negatively correlated with the De Ritis ratio (*p* < 0.05 for all). No significant correlations were found in the healthy control group.

When all participants were analyzed collectively, APRI was positively correlated with fasting BA and NICU admission, and negatively correlated with the De Ritis ratio, gestational age at delivery, and birth weight (Table 3).

**Table 3:** Correlation between APRI and clinical parameters in pregnant women with intrahepatic cholestasis and healthy controls

Variable	Mild ICP (*n* = 62)	Severe ICP (*n* = 57)	Healthy (*n* = 119)	All Pregnancies (*n* = 238)
Fasting BA	*r* = 0.145 *p* = 0.262	r = 0.476** *p* < 0.001	*r* = 0.021 *p* = 0.823	r = 0.660** *p* < 0.001
De Ritis ratio	r = − 0.494** *p* < 0.001	r = − 0.410** *p* = 0.002	*r* = − 0.044 *p* = 0.631	r = − 0.540** *p* < 0.001
Age	*r* = 0.172 *p* = 0.181	*r* = 0.188 *p* = 0.162	*r* = 0.039 *p* = 0.676	*r* = 0.121 *p* = 0.062
BMI (kg/m²)	*r* = 0.084 *p* = 0.516	*r* = − 0.131 *p* = 0.333	*r* = − 0.107 *p* = 0.248	*r* = − 0.010 *p* = 0.873
Birth week	*r* = − 0.167 *p* = 0.194	*r* = − 0.075 *p* = 0.580	*r* = − 0.060 *p* = 0.518	r = − 0.414** *p* < 0.001
Birth weight (g)	r = − 0.325** *p* = 0.010	*r* = − 0.035 *p* = 0.796	*r* = − 0.055 *p* = 0.553	r = − 0.375** *p* < 0.001
NICU admission	r = 0.334** *p* = 0.008	r = 0.549** *p* < 0.001	*r* = 0.048 *p* = 0.607	r = 0.480** *p* < 0.001

*APRI* Aspartate aminotransferase-to-platelet ratio index, *BMI* Body mass index, *NICU* Neonatal intensive care unit

Correlations were calculated using Spearman’s correlation analysis

r: Correlation coefficient

*p* < 0.05 was considered statistically significant

** Statistically significant correlation

### ROC curve analyses

**Fig. 2:**
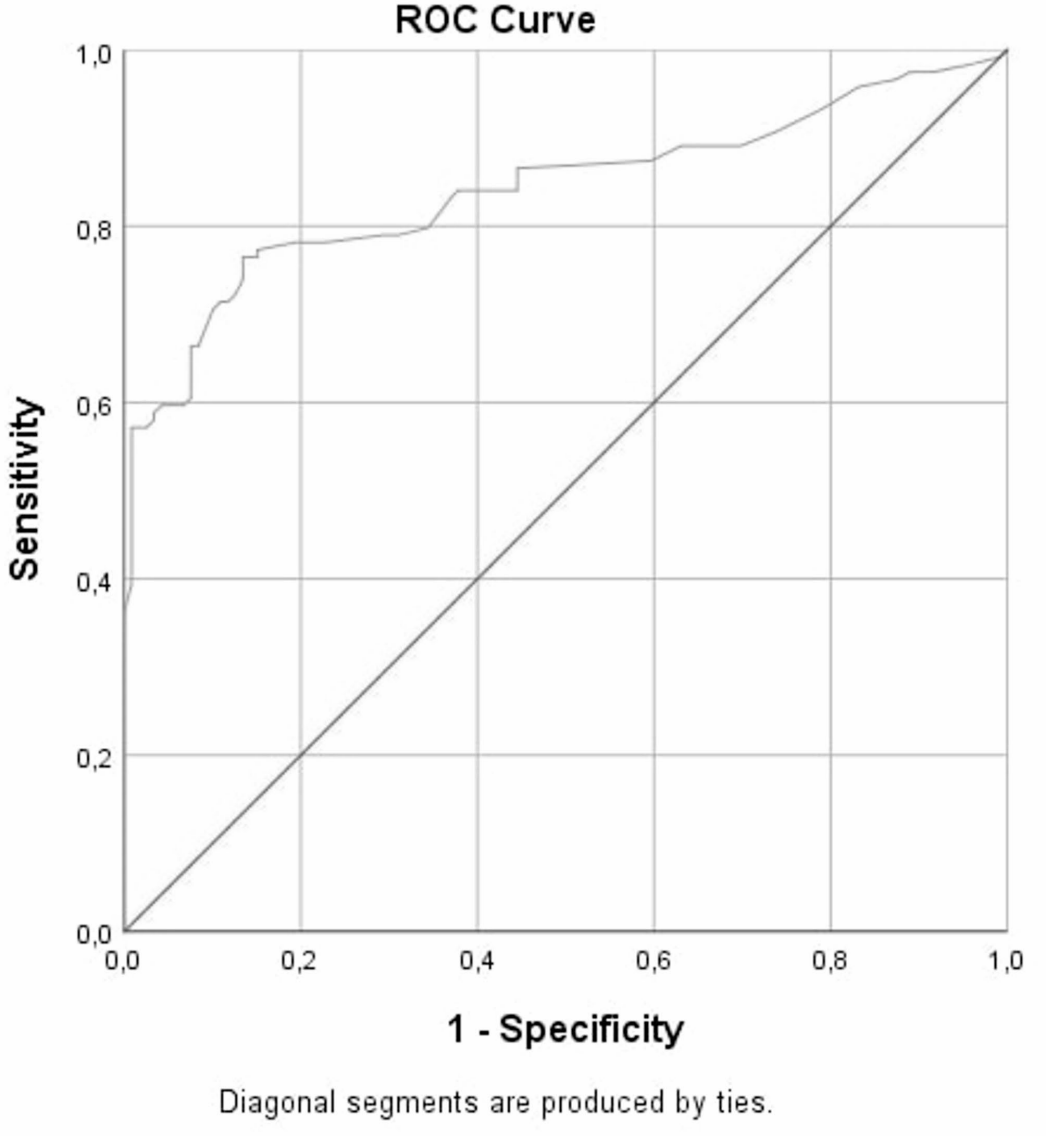
ROC analysis of APRI in ICP and healthy pregnant women. The image you provided has been reviewed. It displays a standard ROC (Receiver Operating Characteristic) curve with: X-axis labeled as “1 - Specificity”, Y-axis labeled as “Sensitivity”, A blue curve representing the performance of APRI in distinguishing ICP from healthy pregnancies, A red diagonal line indicating the reference line of random classification

**Fig. 3:**
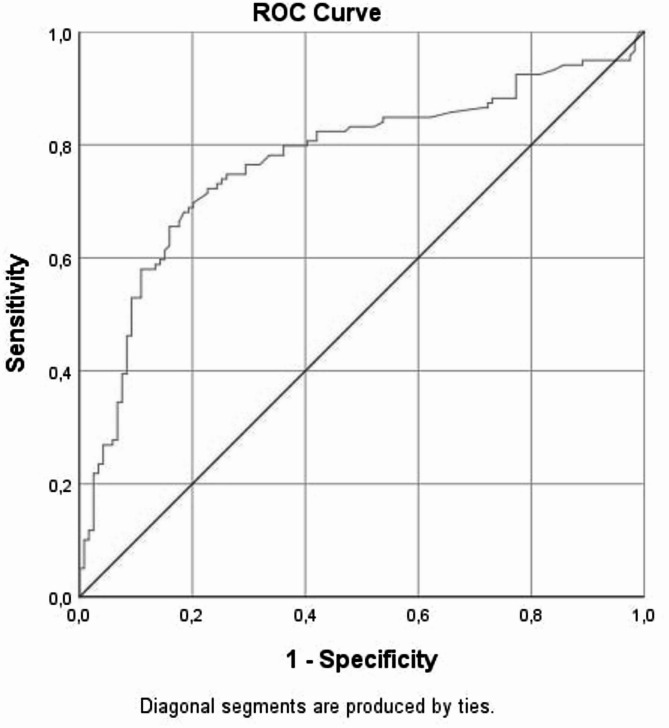
ROC analysis of De Ritis ratio in ICP and healthy pregnant women. The image presents a standard ROC (Receiver Operating Characteristic) curve for the De Ritis ratio. It includes: X-axis labeled as “1 - Specificity”, Y-axis labeled as “Sensitivity”, A blue curve indicating the discriminative performance of the De Ritis ratio in distinguishing ICP from healthy pregnancies, A red diagonal line representing the line of no-discrimination (random chance level). This graphical representation shows the diagnostic accuracy of the De Ritis ratio, with a greater area under the curve indicating stronger predictive power

ROC analysis showed that an APRI value above 0.295 differentiated ICP patients from healthy pregnancies with 78.2% sensitivity and 80.7% specificity (AUC = 0.842, *p* < 0.001). Similarly, a De Ritis ratio below 1.097 was diagnostic for ICP with 76.5% sensitivity and 70.6% specificity (AUC = 0.770, *p* < 0.001) (Figs. 2 and 3; Table 4).

**Table 4:** ROC analysis results of APRI and de Ritis ratio in discriminating intrahepatic cholestasis of pregnancy from healthy pregnancies

Marker	AUC	*p* value	95% CI	Cut-off	Sensitivity (%)	Specificity (%)
APRI	0.842	**< 0.001**	0.789–0.895	0.295	78.2	80.7
De Ritis ratio	0.770	**< 0.001**	0.708–0.833	1.097	76.5	70.6

*AUC* Area under the curve, *CI* Confidence interval, *APRI* Aspartate aminotransferase-to-platelet ratio index

ROC analysis was performed to evaluate the diagnostic performance of APRI and De Ritis ratio in distinguishing ICP from healthy pregnancies

**Fig. 4:**
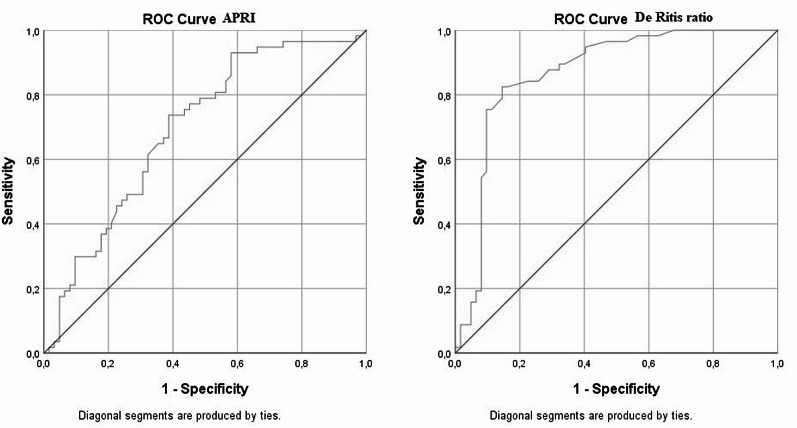
ROC curves of APRI and De Ritis ratio in distinguishing severe from mild intrahepatic cholestasis of pregnancy. This combined ROC graph compares the diagnostic accuracy of APRI and the De Ritis ratio in separating severe ICP cases from mild ones. APRI demonstrated stronger diagnostic performance (AUC = 0.868; cutoff = 0.615; sensitivity = 82.5%; specificity = 83.9%) compared with the De Ritis ratio (AUC = 0.695; cutoff = 0.852; sensitivity = 73.7%; specificity = 61.3%)

To further assess the diagnostic performance of these indices in grading disease severity, a separate ROC analysis was performed comparing severe and mild ICP cases. The APRI showed excellent discriminative ability with an AUC of 0.868, a cutoff value of 0.615, sensitivity of 82.5%, and specificity of 83.9%. In contrast, the De Ritis ratio yielded moderate performance, with an AUC of 0.695, cutoff value of 0.852, sensitivity of 73.7%, and specificity of 61.3% (Table 5). These findings are also illustrated in the combined ROC curve (Fig. 4).

**Table 5:** Receiver operating characteristic (ROC) analysis of APRI and de Ritis ratio in differentiating severe from mild intrahepatic cholestasis of pregnancy

Marker	AUC	*P*-value	95% Confidence Interval	Cutoff	Sensitivity (%)	Specificity (%)
APRI	0.868	< 0.001	0.798–0.937	0.615	82.5	83.9
De Ritis ratio	0.695	< 0.001	0.600–0.789	0.852	73.7	61.3

APRI demonstrated strong discriminative ability (AUC = 0.868), whereas the De Ritis ratio showed moderate performance (AUC = 0.695) in distinguishing severe from mild ICP

*AUC* Area under the curve, *APRI* Aspartate Aminotransferase to Platelet Ratio Index, *CI* Confidence Interval

## Discussion

In this study, we demonstrated that APRI values were significantly elevated in pregnant women with intrahepatic cholestasis of pregnancy (ICP), particularly in those with severe disease. The De Ritis ratio was significantly lower in the ICP groups, and both indices showed significant correlations with fasting bile acid levels and perinatal outcomes. ROC curve analysis indicated that APRI and the De Ritis ratio had good discriminatory ability in distinguishing ICP from healthy pregnancies and in grading disease severity. Additionally, elevated APRI values were associated with increased rates of neonatal intensive care unit (NICU) admission, lower gestational age at delivery, and reduced birth weight. These findings suggest that both APRI and the De Ritis ratio may serve as supportive markers for the diagnosis and severity assessment of ICP.

### First trimester APRI

Several studies have demonstrated that elevated APRI levels in the first trimester are associated with the later development of ICP. For example, Tolunay et al. reported significantly higher first-trimester APRI levels among women who subsequently developed ICP, identifying a diagnostic cut-off of 0.57 (sensitivity 86.5%, specificity 77.3%) [9]. Gok et al. reported similar findings, with a lower cut-off of 0.148 (sensitivity 79.6%, specificity 56.5%) [4], while Konukçu et al. identified a cut-off of 0.16, with a sensitivity of 71% and specificity of 74% [12]. In a study by Kale, a first-trimester APRI cut-off of 0.191 showed moderate diagnostic accuracy (sensitivity 66%, specificity 66%) [13]. Dincgez et al. proposed a cut-off of 0.42, with lower sensitivity (36.6%) but very high specificity (98.3%) [14]. Collectively, these findings suggest that APRI in early pregnancy may be a useful predictor for the subsequent development of ICP.

### Second and third trimester APRI

As pregnancy progresses, APRI levels tend to increase among women with ICP. Studies by Bozbay et al., Peker et al., and Sakcak et al. reported significantly elevated APRI levels in the second and third trimesters among ICP patients compared with healthy controls [11, 15, 16]. The diagnostic performance of APRI was especially notable in the third trimester, with reported cut-off values ranging from 0.098 to 0.13. In our study, third-trimester APRI was significantly elevated in both mild and severe ICP groups compared with controls. ROC analysis identified a cut-off value of > 0.295, with a sensitivity of 78.2% and a specificity of 80.7%, consistent with previous literature.

### APRI and severity of ICP

While many studies have investigated the diagnostic value of APRI in ICP, fewer have evaluated its association with disease severity. Eyisoy et al. found significantly higher APRI values in severe ICP cases compared with mild ones, with a cut-off of 1.06 for predicting severe disease (sensitivity 82%, specificity 72%) [17]. Similarly, our study showed that APRI was significantly higher in severe ICP than in mild cases, with a diagnostic cut-off of > 0.615 (sensitivity 82.5%, specificity 83.9%).

### Relationship between APRI and serum bile acids

Serum bile acids (BA) are considered the standard biomarker for both the diagnosis and severity assessment of ICP. Several studies have reported a positive correlation between APRI and serum BA levels. Cemortan et al., Eyisoy et al., and Tolunay et al. all demonstrated significant associations between APRI and fasting BA concentrations in pregnancies affected by ICP [1, 10, 17]. In line with these findings, our study revealed a strong positive correlation between APRI and fasting BA across all participants, with the strongest correlation observed in the severe ICP group. These results support the potential utility of APRI as an indirect marker of bile acid elevation and disease severity. Similarly, Demir and Sertel recently reported that both first- and third-trimester APRI scores were significantly higher in pregnant women with ICP compared to healthy controls, and that these scores were positively correlated with serum total bile acid levels at diagnosis [18]. These findings are in agreement with our results and further support the role of APRI as a surrogate biomarker for bile acid elevation. However, unlike our study, their analysis did not investigate the relationship between APRI and perinatal outcomes. Our findings thus provide additional evidence that elevated APRI values are not only reflective of biochemical severity but may also be associated with adverse neonatal outcomes.

### De Ritis ratio and its diagnostic role

The AST/ALT ratio, commonly known as the De Ritis ratio, has traditionally been used to differentiate between various liver pathologies [19–21]. In the context of ICP, both Dincgez and Kale reported significantly lower De Ritis ratios in ICP patients compared with healthy controls, highlighting its diagnostic utility with proposed cut-offs of ≤ 1.3 and < 1.07, respectively [14, 15]. Consistent with these reports, our study found that the De Ritis ratio was significantly reduced in ICP, with further declines corresponding to increased disease severity. ROC analysis demonstrated that a De Ritis ratio below 1.09 predicted the presence of ICP, while a cut-off below 0.85 was indicative of severe disease. Moreover, we identified a strong negative correlation between the De Ritis ratio and APRI, particularly within the ICP groups, supporting its specificity in reflecting hepatic dysfunction in ICP.

### Perinatal outcomes

The association between APRI and neonatal outcomes remains a topic of debate. While Karabay et al. found no significant relationship between APRI and neonatal outcomes [22], studies by Sakcak et al. and Eyisoy et al. reported significant correlations between elevated APRI levels and NICU admission [16, 17]. Our findings are consistent with the latter, as we observed a positive correlation between APRI and NICU admission. NICU admission was also significantly more frequent in ICP cases, particularly among those with severe disease. Additionally, we found negative correlations between APRI and both gestational age at delivery and birth weight. First- and fifth-minute Apgar scores were significantly lower in the ICP groups compared with healthy controls. These observations suggest that elevated APRI is associated with adverse neonatal outcomes, particularly in severe ICP cases.

In some of our ICP cases, pruritus was noted in medical records before any increase in serum bile acids or liver enzymes, which is consistent with the observation by Kenyon et al. [23] that pruritus may precede biochemical abnormalities. This finding highlights the importance of early clinical recognition, especially in settings where bile acid results may be delayed.

Our study classified disease severity based solely on fasting serum bile acid levels. While this approach may increase specificity, previous studies have indicated that relying exclusively on fasting values can reduce sensitivity (< 30%) and may underestimate peak bile acid levels in some severe cases [24].

Although most patients underwent close fetal monitoring including weekly NST and biweekly BPP, the benefit of routine umbilical artery Doppler surveillance in isolated ICP remains uncertain, as previously noted by Zimmermann et al. [25].

Future multicenter studies and meta-analyses are warranted to validate these findings and enhance their generalizability across diverse populations.

### Strengths and limitations of the study

One of the strengths of this study is the classification of ICP patients into subgroups based on disease severity. This allowed for the evaluation of the relationship between disease severity and APRI—an easily calculable, cost-effective parameter that does not require additional testing—thereby enhancing the clinical applicability of the findings.

Additionally, the inclusion of a large control group of healthy pregnant women increased the statistical power of the study.

This study has several limitations. First, its retrospective, singlecenter design may limit the generalizability of the findings. Second, only fasting bile acid levels at a single time point were analyzed; peak or serial measurements were not performed, which might underestimate disease severity. Finally, fetal monitoring protocols were individualized rather than standardized, which may limit the interpretation of perinatal outcomes.

## Conclusions

Preventing pregnancy-related complications remains a crucial goal in obstetric care. Early prediction and diagnosis of ICP, combined with appropriate monitoring and treatment, can reduce the risk of severe perinatal complications and minimize maternal and fetal morbidity and mortality. APRI and the De Ritis ratio—both inexpensive, noninvasive, and easily accessible—may serve as useful tools for predicting ICP and identifying severe cases in clinical practice.

## Data Availability

The datasets used and analysed during the current study available from the corresponding author on reasonable request.

## Abbreviations

ALT: Alanine aminotransferase
APRI: Aspartate aminotransferase to platelet ratio index
AST: Aspartate aminotransferase
AUC: Area under the curve
BA: Bile acid
BMI: Body mass index
CS: Cesarean section
GDM: Gestational diabetes mellitus
GA: Gestational age
HELLP: Hemolysis, elevated liver enzymes, and low platelet count
ICP: Intrahepatic cholestasis of pregnancy
NICU: Neonatal intensive care unit
PE: Preeclampsia
PLT: Platelet
ROC: Receiver operating characteristic
UDCA: Ursodeoxycholic acid
USG: Ultrasonography
WBC: White blood cell
